# Immunogenicity of Different Forms of Middle East Respiratory Syndrome S Glycoprotein

**Published:** 2019

**Authors:** T. A. Ozharovskaia, O. V. Zubkova, I. V. Dolzhikova, A. S. Gromova, D. M. Grousova, A. I. Tukhvatulin, O. Popova, D. V. Shcheblyakov, D. N. Scherbinin, A. S. Dzharullaeva, A. S. Erokhova, M. M. Shmarov, S. Y. Loginova, S. V. Borisevich, B. S. Naroditsky, D. Y. Logunov, A. L. Gintsburg

**Affiliations:** Federal Research Centre of Epidemiology and Microbiology named after Honorary Academician N. F. Gamaleya, Ministry of Health of Russian Federation, Gamaleya Str. 18, 123098, Moscow, Russia; «48 Central Research Institute», Ministry of Defense of Russian Federation, Oktyabrskaya Str. 11, 141306 , Sergiev Posad, Russia

**Keywords:** Middle East respiratory syndrome, MERS, MERS-CoV, glycoprotein, adenoviral vector, immunity

## Abstract

The Middle East respiratory syndrome coronavirus (MERS-CoV) was identified in 2012 during the first Middle East respiratory syndrome (MERS) outbreaks. MERS-CoV causes an acute lower-respiratory infection in humans, with a fatality rate of ~35.5%. Currently, there are no registered vaccines or means of therapeutic protection against MERS in the world. The MERS-CoV S glycoprotein plays the most important role in the viral life cycle (virus internalization). The S protein is an immunodominant antigen and the main target for neutralizing antibodies. In the present study, the immunogenicities of five different forms of the MERS-CoV S glycoprotein were compared: the full-length S glycoprotein, the full-length S glycoprotein with the transmembrane domain of the G glycoprotein of VSV (S-G), the receptor-binding domain (RBD) of the S glycoprotein, the membrane-fused RBD (the RBD fused with the transmembrane domain of the VSV G glycoprotein (RBD-G)), and the RBD fused with Fc of human IgG1 (RBD-Fc). Recombinant vectors based on human adenoviruses type 5 (rAd5) were used as delivery vehicles. Vaccination with all of the developed rAd5 vectors elicited a balanced Th1/Th2 response in mice. The most robust humoral immune response was induced after the animal had been vaccinated with the membrane-fused RBD (rAd5-RBD-G). Only immunization with membrane forms of the glycoprotein (rAd5-S, rAd5-S-G, and rAd5-RBD-G) elicited neutralizing antibodies among all vaccinated animals. The most significant cellular immune response was induced after vaccination of the animals with the full-length S (rAd5-S). These investigations suggest that the full-length S and the membrane form of the RBD (RBD-G) are the most promising vaccine candidates among all the studied forms of S glycoprotein.

## INTRODUCTION

The Middle East respiratory syndrome (MERS) is an acute inflammatory respiratory infection that was first identified in Saudi Arabia in June 2012. The causative agent of MERS was officially called the Middle East respiratory syndrome coronavirus (MERS-CoV) in 2013 [1, 2]. MERS-CoV is a single-stranded RNA virus belonging to the Coronaviridae family, genus Betacoro navirus. Dromedary camels are the natural reservoir of MERS-CoV; viral transmission to humans occurs during consumption of unpasteurized camel milk; airborne transmission is also possible [3-5].

To date, a total of 2,260 laboratory-confirmed cases of MERS-CoV infection have been reported, including 803 deaths [6, 7]. Due to its high mortality rate (~ 35.5%) [6], combined with a wide distribution of the reservoir and absence of effective preventive drugs or treatment, WHO experts classify MERS-CoV as a virus with the potential to cause a pandemic [8]. Therefore, vaccine development is necessary in order to stave off such a pandemic.

The main protective MERS-CoV antigen is the S glycoprotein presented as a trimer on the virus surface. The S glycoprotein plays an important role in the virus’ internalization into the cell [9]. The S glycoprotein is subdivided into two subunits: the S1 subunit containing the receptor-binding domain (RBD), and the S2 subunit responsible for the fusion of the virus and cell membrane [10-13]. These features make the S protein an important target for MERS-CoV vaccine development [14-17]. The RBD of the MERS-CoV S glycoprotein is a key target for the development of preventive and therapeutic means against MERS [18-20], because the RBD mediates the interaction between MERS-CoV and the receptor DPP4 on the cell surface.

In order to develop an effective MERS-CoV vaccine, it is important to understand which form of the glycoprotein to include in the vaccine to provide protection against MERS. There are data that demonstrate the immunogenicity of various forms of the MERS-CoV glycoprotein [21-25]. However, the question of which form is preferable for a vaccine remains open, since an antigen panel has not been tested under the same conditions. In order to address this knowledge gap, we constructed five recombinant vectors based on human adenovirus type 5 (rAd5) expressing different forms of the MERS-CoV S glycoprotein:

– the full-length S glycoprotein localized in the endoplasmic reticulum (ER);

– two secreted variants of the receptor binding domain of the MERS-CoV S glycoprotein containing the alkaline phosphatase leader peptide (the RBD and the RBD fused with Fc of human IgG1 to increase stability and immunogenicity [18,19]); and

– two transmembrane (TM) forms localized in the plasma membrane of the cell: either the full-length S or the RBD with the TM domain of the vesicular stomatitis virus (VSV) G glycoprotein. Because the full-length S glycoprotein is localized in the ER [26], we constructed an S-G variant with a deleted ER localization signal.

We chose a platform based on the recombinant viral vectors rAd5 for delivering the S glycoprotein, because such vectors can efficiently deliver the transgene to multiple cell types [27, 28], their genome has been fully characterized, they are able to grow to high titers [29], and they can induce a strong humoral and cellular immune response [30, 31].

The present study compares the humoral and cellular immune responses induced by vaccination of mice with rAd5 carrying different forms of the MERS-CoV S glycoprotein.

## EXPERIMENTAL

**Cell lines**

The HEK293 and A549 cell lines were obtained from the Russian collection of vertebrate cell lines (Russia). The HEK293T and Vero E6 cells were obtained from ATCC (USA). All cells were cultured in DMEM (Dulbecco’s modified Eagle medium) supplemented with 10% fetal bovine serum at 37°C with 5% CO_2_.

**Construction of recombinant adenoviral particles expressing different forms of the MERS-CoV S glycoprotein**

The MERS-CoV S glycoprotein amino acid sequences of strains (2015–2017) were obtained from the NCBI database [32]. The consensus sequence of the MERS-CoV S glycoprotein was made on the basis of amino acid sequences using the Geneious® 10.2.3 software. The nucleotide sequences of different glycoprotein forms were optimized for expression in mammalian cell lines and synthesized by Evrogen JSC (Russia). Five recombinant plasmids (pAd5-S, pAd5- S-G, pAd5-RBD, pAd5-RBD-G, and pAd5-RBD-Fc) were generated. rAd5 was obtained according to the procedure described previously [33].

**Generation of lentiviral particles pseudotyped with MERS-CoV S glycoprotein (pseudoviruses)**

HEK293T cells were seeded in 15-cm culture Petri dishes and co-transfected with three plasmids (pCMVΔR8,2; pLV-CMV-EGFP; pCMV-MERS-CoV-S) to obtain pseudoviruses. Seventy-two hours later, the supernatants were collected, filtered, divided into aliquots, and stored at –80°C. Vero E6 cells were used for titrating the pseudoviruses. The titer of the pseudotyped virus was determined in terms of focus-forming units (FFUs).

**Evaluating the expression of different forms of the MERS-CoV S glycoprotein by western blotting**

HEK293 cells were seeded in 35 mm Petri dishes and incubated overnight to 70% confluence. Then, rAd5 were added to the cells at 100 PFUs/cell. rAd5-null was used as a control virus. After 24 h, the expression of different forms of the MERS-CoV S glycoprotein was evaluated by western blotting using S-specific antibodies (40069-RP02, Sino Biological, China) and antibodies specific to rabbit IgG (NA934V, GE, Great Britain). Expression of the membrane-fused forms of the glycoprotein (S, S-G, RBD-G) was detected in cell lysates prepared using the Cell Culture Lysis Reagent (Promega, USA). Expression of the secreted versions of the glycoprotein (RBD, RBD-Fc) was evaluated in the culture medium. Lysate samples were loaded onto wells (10 μg of total protein in a volume of 10 μl/well). Samples of the medium were loaded in volume 10 μl/well.

**Laboratory animals**

All animal experiments were performed in strict accordance with the recommendations of the National Standard of the Russian Federation (GOST R 53434–2009; Principles of Good Laboratory Practice). Six-week-old female C57/BL6 mice (18–20 g) were obtained from the Pushchino Breeding Facility (Russia). The mice had free access to water and food. The mice were housed in an ISOcage system (Tecniplast, Italy).

**Immunization and serum samples collection**

The mice were randomly distributed into groups (n = 5 per group for the analysis of the humoral immune response and n = 9 per group for the analysis of the T-cell response) and intramuscularly vaccinated with the obtained recombinant adenoviral particles at a dose of 108 v.p./mouse in a total volume of 0.1 mL. Serum specimens were collected on day 21 post-vaccination for detection of S-specific IgG antibodies.

**Determination of antibody titers in mouse serum samples using enzyme-linked immunosorbent assay (ELISA)**

Glycoprotein-specific antibody titers in mouse serum samples were determined by enzyme-linked immunosorbent assay (ELISA). The following recombinant proteins were used for analysis: the S glycoprotein (40069- V08B; Sino Biological) and the RBD (40071-V08B1; Sino Biological). Non-specific antibody binding sites were blocked with PBS with 0.1% Tween 20 (PBST) containing 5% fat-free milk (A0830; AppliChem, Spain). The serum samples were titrated with two-fold serial dilutions in PBST containing 3% fat-free milk. The following anti-mouse IgG horseradish peroxidase-conjugated secondary antibodies were used for detection: for the total IgG titer, NXA931 (GE Healthcare, USA); for IgG1, ab97240 (Abcam, UK); for IgG2a, ab97245 (Abcam, UK); for IgG2b, ab97250 (Abcam, UK); and for IgG3, ab97260 (Abcam, UK). A tetramethylbenzidine solution (Research Institute of Organic Semiproducts and Dyes, Russia) was used as a visualizing reagent. The reaction was stopped by adding 1M H_2_SO_4_, and the optical density was measured at 450 nm (OD_450_) using a Multiscan FC spectrophotometric plate reader (Thermo Fisher Scientific). The IgG titer was determined as the maximum serum dilution in which the OD_450_ value of a serum sample from an immunized animal exceeded that of the control animal serum sample more than twofold.

**Pseudovirion-based neutralization assay (PsVNA)**

The pseudovirion-based neutralization assay (PsVNA) was performed as described previously [34]. Briefly, heat-inactivated serum samples were diluted 1:10, 1:40, 1:160, and 1:640. These samples were then mixed with an equal volume of DMEM containing 105 FFUs/ml of the pseudovirions. The mixture was incubated at 37°C for 1 h, then inoculated onto a Vero E6 cell monolayer and incubated at 37°C for 42 h. The number of EGFP fluorescent cell focuses was counted. The pseudovirion neutralization titer of serum samples from an immunized animal was determined as the maximum dilution where 50% reduction of EGFP fluorescent cell focuses compared with the serum samples of intact (non-immunized) animals was determined.

**Analysis of T-cell response (lymphocyte proliferation assay)**

Mice were euthanized on day 8 post-vaccination, and their spleens were collected. The spleens were homogenized by passage through a 100 μm sieve in sterile PBS. Splenocytes were isolated by Ficoll (1.09 g/mL; PanEco, Russia) density gradient centrifugation (800 *g *for 30 min). For T-cell proliferation assay, the splenocytes were stained with carboxyfluorescein using a succinimidyl ester (CFSE) tracer kit (Invitrogen, USA) according to the procedure described previously [35]. The cells were seeded in 96-well plates (2 × 10^5^ cells/well) in a complete RPMI1640 medium re-stimulated with the recombinant MERS-CoV S protein (40071-V08B1; Sino Biological) at 1 μg/well. After 3 days, the cells were harvested, washed with PBS, stained with antibodies specific to CD3, CD4, and CD8: allophycocyanin (APC)-labelled anti-CD3, APC–Cy7-labelled anti-CD8, and phycoerythrin-labelled anti-CD4 (BD Biosciences, USA), and fixed in 1% paraformaldehyde. Proliferating CD4+ and CD8+ T lymphocytes were determined in the cell mixture using a BD FACS Aria III flow cytometer (BD Biosciences). The resulting percentage of proliferating cells (X) was determined using the formula X = %st – %, where %st is the percentage of proliferating cells after splenocyte re-stimulation with the recombinant MERS-CoV S glycoprotein, and % is the percentage of proliferating cells in the absence of splenocyte re-stimulation (intact cells).

**Analysis of interferon gamma (IFN-γ) production**

Splenocytes were isolated on day 15 post-vaccination using the procedure described above. The cells were seeded in 96-well plates (2 × 10^5^ cells/well) in a RPMI1640 medium, followed by re-stimulation with recombinant MERS-CoV S (40071-V08B1; Sino Biological) at a concentration of 1 μg/well. Forty-eight h post-treatment, the culture supernatants were collected. The concentration of IFN-γ in the supernatants was measured by ELISA using a commercial kit (mouse IFN-γ ELISA kit; Invitrogen) according to the manufacturer’s instructions. The increase in IFN-γ concentration was determined using the formula X = Cst /Cint, where X is the fold increase in IFN-γ concentration, Cst is the IFN-γ concentration in the medium from the stimulated cells (pg/ml), and Cint is the IFN-γ concentration in the medium from the non-stimulated (intact) cells (pg/ml).

**Statistical analysis**

The statistical analysis was performed using the GraphPad 7.0 software (GraphPad Software, USA). When analysing data from unpaired samples, either the Student’s t-test for independent samples or the Mann–Whitney U*-*test was used depending on the data distribution normality. Distribution normality was determined using the generalized D’Agostino–Pearson test.

## RESULTS

**Generation of rAd5 vectors**

**Fig. 1:**
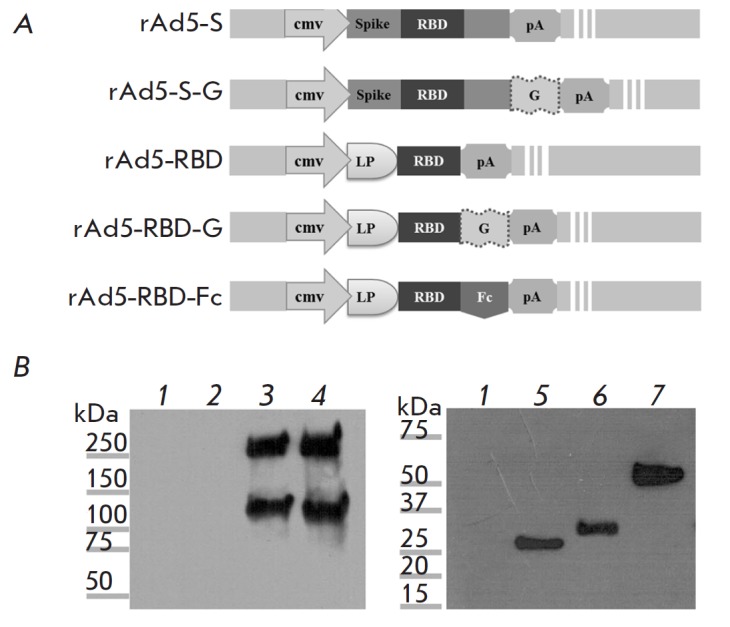
*A *– Schematic structures of target transgenes in the rAd5-S, rAd5-S-G, rAd5-RBD, rAd5-RBD-G, and rAd5-RBD-Fc genomes. Cmv – a promoter of the E1 region of human cytomegalovirus; G – the gene of the G glycoprotein of the vesicular stomatitis virus; LP – the leader-peptide sequence directing protein secretion; pA – polyadenylation signal; RBD – the receptor-binding domain of MERS-CoV S glycoprotein; Spike – MERS CoV S glycoprotein. *B *– Western blot analysis of the MERS-CoV S glycoprotein variants expressed by each rAd5. Lane *1 *– lysate from control Ad5-null cells; lane *2 *– intact cells; lane *3 *– lysate from rAd5-S cells; lane *4 *– lysate from rAd5-S-G cells; lane *5 *– the medium from the rAd5- RBD cell culture; lane *6 *– lysate from rAd5-RBD-G cells; lane *7 *– the medium from the rAd5-RBD-Fc cell culture

In order to determine and compare the immunogenicities of different forms of the MERS-CoV S protein, we constructed five rAd5 vectors: rAd5-S, rAd5-S-G, rAd5-RBD, rAd5-RBD-G, and rAd5-RBD-Fc. The schemes for the target transgenes in the rAd5 genomes are shown in *Fig. 1A*. Expression levels of different forms of the MERS-CoV S glycoprotein were evaluated by western blot analysis (*Fig. 1B*). In the samples of the full-length glycoprotein (rAd5-S and rAd5-S-G), the S protein was detected as two polypeptides (*Fig. 1B*), with the upper band representing the glycosylated form of the S protein (~230–250 kDa), and the lower band (~100 kDa) representing the S1 subunit resulting from S-protein cleavage by host-cell proteases. The molecular weights of the bands were higher than those calculated according to the nucleotide sequences, being indicative of tentative protein glycosylation [14, 16, 36, 37, 38]. A single polypeptide specifically recognized by the antibody was detected in the RBD (RBD, RBD-G and RBD-Fc) samples (*Fig. 1B*), its molecular weight being ~25 kDa, ~30 kDa, and ~55 kDa, respectively. The molecular weights of the polypeptides based on the RBD corresponded to the calculated weights.

**rAd5 expressing different MERS-CoV S glycoprotein variants induce a humoral immune response**

**Fig. 2:**
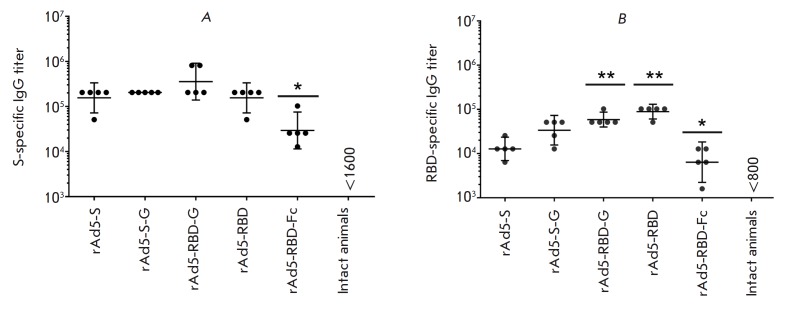
Glycoprotein-specific IgG titers in the blood serum of immunized animals. The figure shows IgG titers: (*A*) specific to the MERS-CoV S glycoprotein and (*B*) specific to the RBD. Scatter plots show the geometric mean titer (GMT) and 95% confidence interval (CI) for each group (n = 5 mice/group). Asterisks indicate significant intergroup differences in IgG titers. * *p * < 0.05, rAd5-RBD-Fc is compared with other groups; ** *p * < 0.05, rAd5-RBD, and rAd5-RBD-G are compared with other groups, except for rAd5-RBD and rAd5-RBD-G (the Mann–Whitney U test)

Mice were intramuscularly immunized with single doses of rAd5-S, rAd5-S-G, rAd5-RBD, rAd5-RBD-G, and rAd5-RBD-Fc (108 v.p. per mouse). Serum samples were collected three weeks after the immunization, and the titers of antibodies specific to S protein and RBD were analyzed (*Fig. 2*). No glycoprotein-specific IgG was detected in the serum samples from mice in the control groups (non-immunized mice and those immunized with rAd5-null). The highest titer of IgG specific to S glycoprotein was detected in the group immunized with rAd5-RBD-G [geometric mean titer (GMT) was 356,055; the 95% confidence interval (CI) was 139,042–911,772]. The lowest titer of IgG specific to the S glycoprotein was observed in the group immunized with rAd5-RBD-Fc [GMT: 29,407; 95% CI: 11,455–75,492] (*Fig. 2A*). ELISA for RBD-specific IgG antibodies showed that rAd5-RBD-G [GMT: 89,144; 95% CI: 60,665–130,994] and rAd5-RBD [GMT: 58,831; 95% CI: 40,024–86,424] were the constructs with the highest immunogenicity, while rAd5-RBD-Fc [GMT: 6,400; 95% CI: 2,230–18,364] had the lowest immunogenicity. No significant differences in RBD-specific IgG titers were detected between rAd5-RBD and rAd5- RBD-G (*Fig. 2B*).

**Fig. 3:**
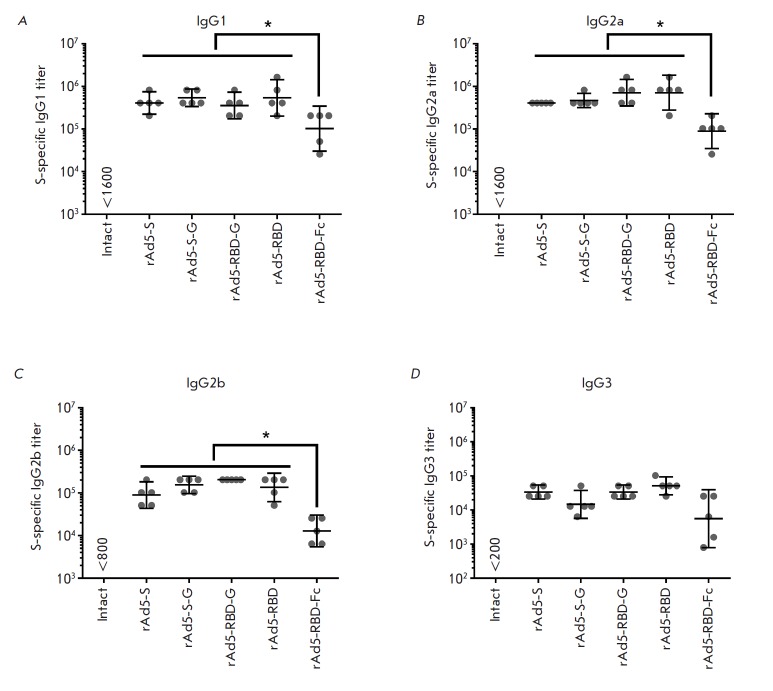
Analysis of IgG antibody isotypes in mice after immunization with rAd5 expressing different forms of the MERS-CoV S glycoprotein. The figure shows the titers of IgG1 (*A*), IgG2a (*B*), IgG2b (*C*), and IgG3 (*D*) specific to the MERS-CoV S glycoprotein in the serum samples of immunized animals. Scatter plots show the geometric mean titer (GMT) and 95% confidence interval (CI) for each group (n = 5). Asterisks indicate significant intergroup differences in IgG titers. * *p * < 0.05, Mann–Whitney U test

There are four IgG isotypes known in mice to be responsible for identification and clearance of many antigens: IgG1, IgG2a, IgG2b, and IgG3 [39]. Determination of the titers of IgG isotypes three weeks post-immunization showed that all four IgG isotypes were detected in all vaccinated animals (*Fig. 3*). For the IgG1, IgG2a, and IgG2b isotypes in immunized animals, titers were as follows for each group: rAd5-S (GMT: 409,600, 409,600, and 89,144, respectively); rAd5-S-G (GMT: 54,0470, 470,506, and 155,209, respectively); rAd5-RBD (GMT: 540,470,713,155, and 135,118, respectively); and rAd5-RBD-G (GMT: 356,578, 713,155, and 204,800, respectively). We observed no significant intergroup difference in isotype titers. Following vaccination with rAd5-RBD-Fc, IgG1, IgG2a, and IgG2b, the titers were significantly lower than those in the other groups (GMT:102,400, 89,144, and 12,800, respectively). The IgG3 titers did not differ significantly between the groups (rAd5-S, rAd5-S-G, rAd5-RBD, rAd5-RBD-G, and rAd5-RBD-Fc; GMT: 33,779, 14,703, 51,200, 33,779, and 5572, respectively). Hence, according to these findings, the IgG1 and IgG2a isotypes make the greatest contribution to the total titer of glycoprotein-specific IgG.

**rAd5 expressing membrane forms of the MERS-CoV S glycoprotein elicit the production of neutralizing antibodies in mice**

**Fig. 4:**
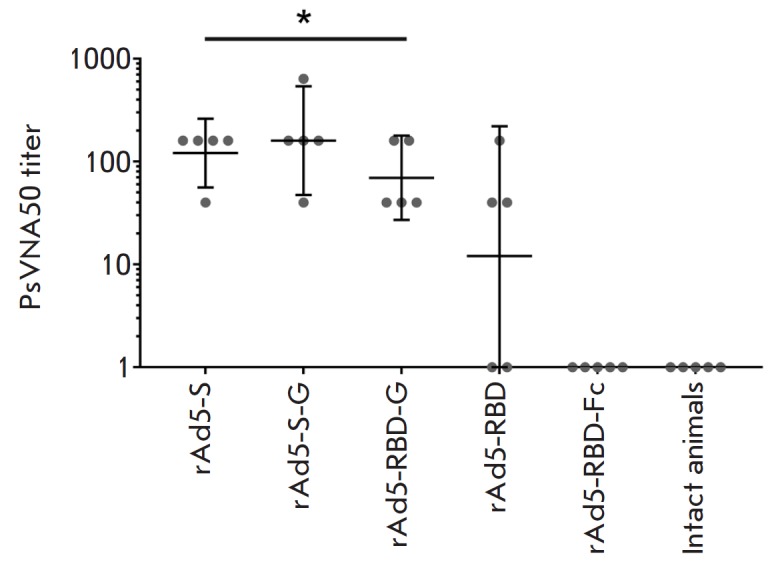
The titers of neutralizing antibodies in the blood serum of immunized animals. The neutralization assay was performed using lentiviral particles pseudotyped with MERS-CoV S glycoprotein. Scatter plots show the geometric mean titer (GMT) and 95% confidence interval (CI) for each group (n = 5). Asterisks indicate no significant intergroup differences in the titers of neutralizing antibodies. * *p * > 0.05, Mann–Whitney U test

Determination of the titers of neutralizing antibodies (in the pseudovirion-based neutralization assay) showed that all mice immunized with rAd5-S, rAd5- S-G, and rAd5-RBD-G generated neutralizing antibodies (*Fig. 4*) with the GMT of 1:121, 1:160 and 1:70, respectively; no significant differences in PsVNA titers were observed (*p *> 0.05). In the rAd5-RBD group, neutralizing antibodies were detected only in three mice while no neutralizing antibodies were detected in the rAd5-RBD-Fc group and in intact animals. Hence, the results of the conducted experiment showed that only immunization with rAd5 expressing the membrane forms of the glycoprotein (S, S-G, RBD-G) leads to the generation of neutralizing antibodies.

**rAd5 expressing the MERS-CoV S protein variants elicit the T-cell response**

**Fig. 5:**
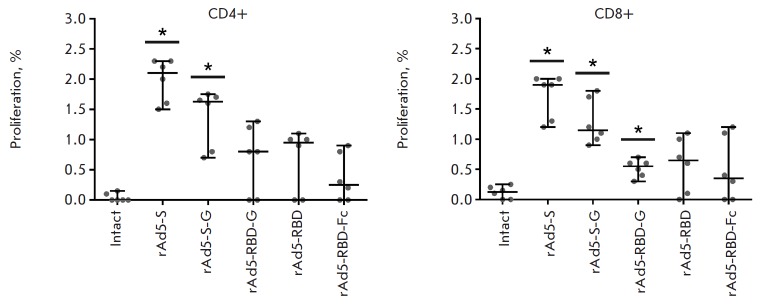
The study of lymphoproliferative activity of splenocytes in immunized mice. The figure shows the levels (%) of proliferating CD4+ and CD8+T cells re-stimulated by the MERS-CoV S protein on day 8 post-vaccination. Scatter plots show the median lymphoproliferative activity of re-stimulated cells (%) with 95% CIs for each group from one representative experiment (n = 6 mice/group). Asterisks indicate significant differences in the percentage of proliferating cells between vaccinated and intact animals. * *p * < 0.05, Mann–Whitney U test

**Fig. 6:**
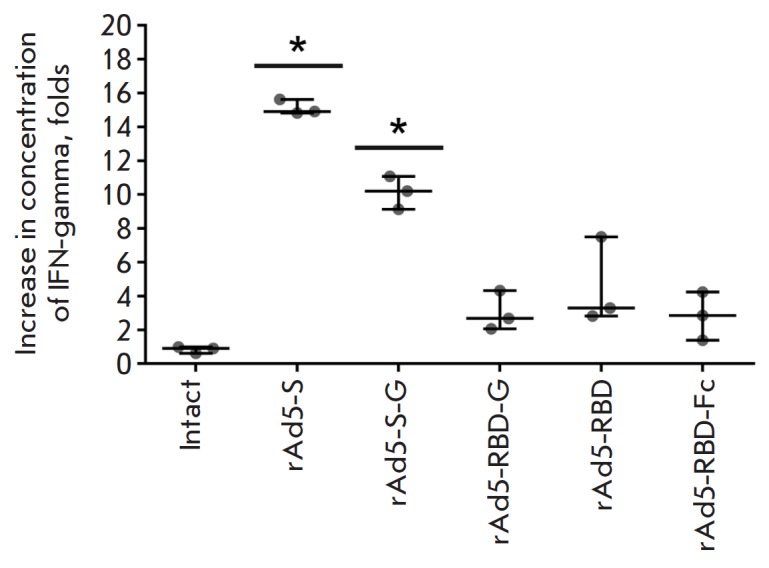
The increase in the concentration of IFN-γ in the media of splenocytes from immunized mice after re-stimulation with the recombinant full-length MERS-CoV S protein. Scatter plots show the median (95% CI) increase (fold change) in IFN-γ production following re-stimulation for each group from one representative experiment (n = 3 mice/group). Asterisks indicate significant differences in IFN-γ production between the cells taken from vaccinated and intact animals. * *p * < 0.05, Student’s t-test for independent samples

The post-vaccination cellular immune response was evaluated using two methods: according to the number of proliferating T cells and according to IFN-γ production by T cells in response to glycoprotein re-stimulation. The full-length MERS-CoV S protein was used for re-stimulation, since it contains the largest number of epitopes and is present in MERS-CoV particles. The proliferation assay of CD4+ cells on day 8 post-vaccination (*Fig. 5*, left-side panel) showed that the highest lymphoproliferative activity was observed in the rAd5-S group (2.10%), while the lowest one was detected in the rAd5-RBD-Fc group (0.25%). Significant differences in the lymphoproliferative response of CD4+ cells between the groups of immunized and intact animals were observed in the rAd5-S (2.10%) and rAd5-S-G (1.63%) groups. CD8+ cells proliferation assay (*Fig. 5*, right-side panel) showed that the highest lymphoproliferative response was observed in the rAd5-S group (1.90%), while the lowest one was detected in the rAd5-RBD-Fc group (0.35%). Significant differences in the lymphoproliferative response of CD8+ cells between the groups of immunized and intact animals were observed in the rAd5-S (1.90%), rAd5-S-G (1.15%), and rAd5-RBD (0.55%) groups. An analysis of IFN-γ production by splenocytes after the MERS-CoV S glycoprotein re-stimulation also showed that the strongest cellular immune response developed in groups of animals immunized with rAd5-S and rAd5-S-G (*Fig. 6*): IFN-γ secretion increased as compared to that for intact cells 15.12 ± 0.43-fold and 10.14 ± 0.97-fold, respectively.

## DISCUSSION

Currently, there are no specific prophylactic or therapeutic agents against the Middle East respiratory syndrome in the world. Intensive research focusing on the development of vaccines against this disease is being conducted in the USA, Germany, South Korea, and other countries [40, 41]. Several candidate vaccines based on MERS-CoV glycoprotein are known: recombinant viral vectors based on the recombinant vaccinia virus, adenovirus, measles virus and others; DNA vaccines; combined candidate vaccines based on DNA and recombinant protein; and candidate vaccines based on virus-like particles and recombinant proteins [22, 38, 41-46].

The key in vaccine development is antigen selection. Most of the developed vaccines against MERS are based on the application of different forms of the MERS-CoV glycoprotein (the full-length S, the S1 subunit, and RBD) [14, 15, 16, 18, 22, 24, 47-55], which is the main target for neutralizing antibodies. However, the question that still remains open is which form to choose for the development of an effective vaccine? It is known that the full-length S glycoprotein ensures 100% protection against lethal infection caused by MERS-CoV in animals [44]. However, some authors have expressed concern about the use of full-length MERS-CoV S in the vaccine. Thus, it has been reported that a vaccine based on a full-length glycoprotein of the severe acute respiratory syndrome (SARS) coronavirus (which, like MERS-CoV, belongs to the genus Betacoronavirus) induces immunopathology in the lungs of a vaccinated organism because of the strong antibody response to the SARS-CoV glycoprotein and weak T cell (Th2-skewed) immune response [56, 57].

Glycoprotein modifications were for the most part based on the fact that the receptor-binding domain of the glycoprotein was included in the antigen. Various studies showed immunogenicity of the S1 subunit, the RBD or the RBD fused with Fc of human IgG1 (RBD-Fc) [15, 18, 19, 49, 58]. Studies focused on protection of drugs based on RBD (subunit vaccines) showed that RBD vaccination protected ~ 80% of animals from MERS-CoV, despite the high titers of neutralizing antibodies capable of blocking the interaction between the virus and the DPP4 receptor on the cell surface [42, 59]. The lack of 100% protection seems to be related to the need to develop a cellular immune response, as well as the need to block fusion of the viral and cell membranes, which is mediated by the S2 subunit. Furthermore, application of subunit-based vaccines, as well as inactivated ones, prevents the emergence of a balanced Th1/Th2 response and often leads to the development of Th2-skewed immunity [22, 42]. In the case of MERS, its development can lead to lung immunopathologies [48]. Therefore, it is important to take into account the fact that the vaccine-induced immunity must be Th1/Th2-balanced when developing an anti-MERS vaccine.

Numerous antigens based on the MERS-CoV S glycoprotein have been studied. However, these antigens, under the same conditions (using the same antigen delivery platform), have not been compared directly. In the present study, direct comparison of the immunogenicities of five different forms of the MERS-CoV S glycoprotein under the same conditions was carried out; rAd5 was used for delivery. Strong antibody (mainly IgG1 and IgG2a) and T-cell immune responses developed in animals vaccinated with rAd5 expressing various forms of the MERS-CoV S glycoprotein. Each of the rAd5 variants allowed for the emergence of a balanced Th1/Th2 response, which is one of the key aspects in the development of an anti-MERS vaccine. In a study focused on the intensity of the humoral immune response, the membrane form of the RBD (rAd5-RBD-G) elicited a more powerful IgG response than the other studied forms. It was also demonstrated that only the membrane forms of the MERS-CoV glycoprotein (rAd5-S, rAd5-S-G, and rAd5-RBD-G) stimulate the production of neutralizing antibodies. In the investigation of cellular immune response intensity, the forms of the full-length MERS-CoV glycoprotein (rAd5-S and rAd5-S-G) were characterized by the strongest immunogenicity.

To sum up, the findings obtained in our study suggest that, among all the studied forms of the MERS-CoV S glycoprotein, the full-length S glycoprotein and the membrane form of RBD (RBD-G) are the most promising candidates for inclusion in a vaccine.

## CONCLUSIONS

The immunogenicities of five forms of MERS-CoV S glycoprotein in mice were compared in this study. A platform based on the recombinant adenoviral vectors rAd5 was used for antigen delivery. The studies have yielded the following results:

– The most powerful humoral immune response was observed in animals immunized with the membrane-bound form of the RBD (rAd5-RBD-G);

– Only the membrane forms of MERS-CoV glycoprotein (rAd5-S, rAd5-S-G and rAd5-RBD-G) induced the generation of neutralizing antibodies in all vaccinated mice;

– The most significant cellular immune response developed after immunization of animals with the full-length glycoprotein (rAd5-S); and

– Vaccination of mice with all developed rAd5 vectors elicited a balanced Th1/Th2 response.

## Abbreviations

95% CI: 95% confidence interval
APC: allophycocyanin; DPP4 – dipeptidyl peptidase 4
Fc: fragment crystallizable
FFU: focus-forming units
rAd5: recombinant vector based on adenovirus type 5
RBD: receptor-binding domain of MERS-CoV S glycoprotein
RBD-Fc: receptor-binding domain of MERS-CoV S glycoprotein fused with Fc of human IgG1
RBD-G: receptor-binding domain of MERS-CoV S glycoprotein fused with the transmembrane domain of the VSV G glycoprotein
S: MERS-CoV glycoprotein
S1, S2: domains of MERS-CoV S glycoprotein
S-G: full-length S glycoprotein with the transmembrane domain of the G glycoprotein of VSV
Th: T helper
VSV: vesicular stomatitis virus
MERS: Middle East respiratory syndrome
MERS-CoV: Middle East respiratory syndrome coronavirus
PFU: plaque-forming unit
v.p.: viral particles
GMT: geometric mean titer
IFNγ: interferon gamma
TM: transmembrane domain
SARS: severe acute respiratory syndrome
SARS-CoV: severe acute respiratory syndrome coronavirus
PBS: phosphate-buffered saline
PBST: PBS supplemented with 0.1% Tween 20
ER: endoplasmic reticulum
